# Assessing the construct validity and reliability of the parental perception on antibiotics (PAPA) scales

**DOI:** 10.1186/1471-2458-14-73

**Published:** 2014-01-23

**Authors:** Arwa Alumran, Xiang-Yu Hou, Jiandong Sun, Abdullah A Yousef, Cameron Hurst

**Affiliations:** 1 School of Public Health & Social Work, Queensland University of Technology, Brisbane 4059, Australia; 2 Institute of Health and Biomedical Innovation, Queensland University of Technology, Brisbane 4059, Australia; 3 Health Information Management and Technology Department, College of Applied Medical Sciences, University of Dammam, Dammam, Saudi Arabia; 4 Paediatrics Pulmonary, Department of Paediatrics, College of Medicine, University of Dammam, Dammam, Saudi Arabia; 5 Clinical Epidemiology Unit, Faculty of Medicine, Khon Kaen University, Srinagarind Hospital, Khon Kaen 40002, Thailand; 6 Data Management and Statistical Analysis Center, Faculty of Public Health, Khon Kaen University, Khon Kaen 40002, Thailand

**Keywords:** Antibiotic overuse, Psychosocial, Measurement instrument, Reliability, Validity, Confirmatory factor analysis, Saudi Arabia

## Abstract

**Background:**

The overuse of antibiotics is becoming an increasing concern. Antibiotic resistance, which increases both the burden of disease, and the cost of health services, is perhaps the most profound impact of antibiotics overuse. Attempts have been made to develop instruments to measure the psychosocial constructs underlying antibiotics use, however, none of these instruments have undergone thorough psychometric validation. This study evaluates the psychometric properties of the Parental Perceptions on Antibiotics (PAPA) scales. The PAPA scales attempt to measure the factors influencing parental use of antibiotics in children.

**Methods:**

1111 parents of children younger than 12 years old were recruited from primary schools’ parental meetings in the Eastern Province of Saudi Arabia from September 2012 to January 2013. The structure of the PAPA instrument was validated using Confirmatory Factor Analysis (CFA) with measurement model fit evaluated using the raw and scaled *χ*^2^, Goodness of Fit Index, and Root Mean Square Error of Approximation.

**Results:**

A five-factor model was confirmed with the model showing good fit. Constructs in the model include: *Knowledge and Beliefs, Behaviors, Sources of information, Adherence,* and *Awareness about antibiotics resistance*. The instrument was shown to have good internal consistency, and good discriminant and convergent validity.

**Conclusion:**

The availability of an instrument able to measure the psychosocial factors underlying antibiotics usage allows the risk factors underlying antibiotic use and overuse to now be investigated.

## Background

Antibiotics are helpful in treating bacterial infections and are effective in reducing mortality and morbidity rates worldwide [1]. Since their introduction, antibiotic usage has become very widespread. The increased usage of antibiotics increases the potential for antibiotic overuse and misuse [2-9], including in children [10]. This growing global public health issue needs to be addressed and managed.

The overuse of antibiotics may cause several harmful effects at both the individual, and the community, level. One of the most important individual risk factors is the development of preventable adverse effects (e.g. adverse gastrointestinal effects) [5,11]. These adverse effects represent a more significant issue in children [8]. Community level risk factors are potentially more serious, and include the development of antibacterial resistance and raising the burden of chronic diseases, which leads to an increase in unnecessary expenditure on health services [12-15].

Antibacterial resistance is a growing public health issue worldwide, and represents a risk to both the community and the individual [6-8]. Antibiotic resistance is highly associated with the overuse of antibiotics to treat viral URTIs [1]. With the emergence of antibacterial resistance, it is inadvisable to use antibiotics indiscriminately [16]. Promoting judicious use of antibiotics by parents could protect children from antibacterial resistance, especially in countries where antibiotics can be obtained without prescription.

In many countries, the problem of antibiotic overuse is exacerbated as a result of policy shortfalls, or inadequate regulation on the distribution of antibiotics. In many of these countries, antibiotics can be purchased over-the-counter at pharmacies without a prescription from a doctor. This takes the decision of antibiotic use out of the hands of the medical professional, and places the decision to use antibiotics firmly with consumers and parents.

Antibiotics are often inappropriately used to treat viral infections including most upper respiratory tract infections (URTIs). URTIs are the most common infections around the world [1,15-18]. Most infections of the upper respiratory tract are viral in nature and neither require, or are effectively treated by, antibiotics [19]. The use of antibiotics to treat viral URTIs is considered a misuse of antibiotics [4,20-23]. Parents’ knowledge regarding URTIs and their treatment needs to be assessed in order to develop strategies that may reduce antibiotic overuse in children.

Several factors might cause a community to overuse antibiotics. These include: (1) factors related to policy controls and regulations governing availability of antibiotics; (2) factors related to public consumption such as attitudes, beliefs, knowledge of antibiotic use, and behaviors (e.g. over-the-counter medication and self-medication); (3) patients’ perceptions regarding patient-doctor interaction; and (4) patient satisfaction of experiences with antibiotics [24-30]. In order to measure the factors associated with the public’s patterns of antibiotic use, a valid and reliable instrument able to tease out the psychosocial constructs representing consumers’ attitudes, beliefs and behaviors to antibiotics needs to be available. An extensive literature review concluded that, at this time, there is no validated instrument that measures the factors influencing parents’ use of antibiotics in children, in particular, or for patients, in general [31].

The parental perceptions on antibiotics (PAPA) instrument was developed [32] and has undergone preliminary validation [33] to assess the factors influencing parents’ use of antibiotics in children (especially with URTIs). Both survey items, and the domains in which they were grouped, were developed using a literature review [31,34] and content-validated by an extensive Delphi process using experts’ knowledge about the use of antibiotics [32]. The developed instrument (PAPA scales) needs to undergo further analysis of its psychometric properties to be considered fully valid and reliable for use in future research.

### Construct validation

Construct validity is the extent to which an instrument measures the construct that it is intended to measure. According to Ramaker *et al.* (2002) [35], factor analysis is often used to measure the inter-correlation of the instrument’s components, which subsequently assists in condensing the number of dimensions in the instrument by grouping the related items under the same dimension. Construct validity is achieved when the tool: (1) measures the differences between contrasting groups of participants, (2) reflects the framework hypothesized in a hypothesis testing study, and (3) can undergo a confirmatory factor analysis which adequately establishes that the measurement model fits the actual data [36]. The aim of this study is to investigate the psychometric properties of the PAPA scales and to demonstrate preliminary evidence of construct validity of the PAPA instrument.

## Methods

### Participants

Like many analyses containing a large number of variables, CFA is generally too complex to prospectively power. Typically, little is known about minimal clinical differences and standard deviations associated with both observed and latent variables. Instead, ‘rules of thumb’ are used to guide in the selection of sample size. We used the sample size approach advocated by Comrey and Lee [37]: 100 = fair, 200 = good, 500 = very good, and >1000 = excellent. We were conservative in the selection of sample size in case subgroup analyses were required (e.g. mother and fathers). Given the informal nature of the Comery and Lee [37] approach, it is important to note the effect sizes (i.e. magnitude of loadings and inter-factor correlations) to gauge the contextual importance of parameter estimates in any subsequent analysis.

Participants comprised a sample of parents attending parental meetings in primary schools in the Eastern Province of Saudi Arabia. Of the 1395 people sampled, 1111 (79.6%) completed and returned their survey. Just over half of the participants were mothers (52%); the majority were not trained in health fields (88.8%); more than half of the sample were employed (57.2%); and only 1% were illiterate, with most of the sample having a diploma or a bachelors degree (62.2%). Parents were aged between 19 and 72 years (median = 37; mean = 38.11, SD = 7.7). Geographical background was reported with 44% of the participants stating they were originally from the Eastern Province, while the rest of the participants’ origins were equally distributed among the other provinces, with the exception of the Northern Province, which was represented by only 3.6% of the individuals.

### Measures

The PAPA instrument [33] is a 32-item instrument that aims to measure the psychosocial factors influencing the overuse of antibiotics in children, especially with upper respiratory tract infections. Items in the instrument are available in Additional file 1. Depending on the nature of the item, parents were asked to rate on a 5-point Likert scale ranging from *strongly disagree* to *strongly agree* or from *never* to *always.* The development, translation, and preliminary validation of the PAPA scales are reported elsewhere [32,33].

The questionnaire measures the following criteria: (1) parents’ demographic characteristics such as gender, number of girls and boys in the family, health training, age, employment status and education levels, geographical background, when they moved to the Eastern Province, and monthly income; (2) child health-related history including the number of cold episodes and antibiotics (courses) used for the youngest child during the last year (ranging from never to more than 6 times a year), and whether any of the children in the family has ever had a serious infectious disease or a chronic disease; and (3) items relating to parents’ psychosocial factors influencing the parental use of antibiotics including knowledge and beliefs, behaviors, adherence, seeking information, awareness about antibiotics resistance, and parents’ perception about doctors’ prescribing behavior.

#### *Knowledge and beliefs*

The knowledge and beliefs scale includes 10 items that measure the extent of parents’ knowledge and beliefs with regards to antibiotics use. Knowledge and beliefs items include questions such as: measuring the parents’ perceptions regarding *the necessity to use an antibiotic for: the common cold* [KB1]*, and/or a sore throat* [KB2]. This construct shows good internal consistency in this sample (Cronbach’s α = 0.836).

#### *Behaviors*

This 5-item scale assesses the behaviors of parents with regards to the use of antibiotics. Most of the behavior-related questions are about past experiences such as: *In the past, I have stopped giving my child an antibiotic because my friends/family advised me to* [B5]. This construct shows good internal consistency in this sample (Cronbach’s α = 0.771).

#### *Seeking information*

This 7-item construct assesses the sources parents use to get their health-related information such as: *nurses and/or other allied health professionals* [SI1]*.* The Seeking information scale shows good internal consistency in this sample (Cronbach’s α = 0.834).

#### *Adherence*

This 5-items subscale assesses the level of parents’ adherence to specific doses of antibiotics in their children. It is represented by questions such as: *skipping one or two antibiotic doses doesn’t make much difference* [AD1]. This construct shows good internal consistency in this sample (Cronbach’s α = 0.765).

#### *Awareness about antibiotics resistance*

This is a 5-item factor that assesses the parents’ awareness about antibiotic resistance. It includes items such as: *antibiotics can be harmful to one’s health* [ABR1]*.* The internal consistency in this sample for this construct appears to be moderate (Cronbach’s α = 0.462).

The *Parents’ perception regarding doctors’ prescribing behaviors’* (PPD) was also initially included in the instrument. This factor was included to measure how parents perceived the prescribing behavior of the doctor (e.g. Insufficiently forthcoming with a prescription for antibiotics). The PPD factor had adequately high loadings in the EFA analysis to warrant it’s consideration in the CFA [33]. However, in our initial CFA (the six factor instrument), the PPD items loaded somewhat lower than in the EFA, although they were still statistically significant (p < 0.001). The main difficulty presented in the initial six-factor measurement model was that PPD factor was not associated with any of other factor in the measurement model. This implies the original six-factor instrument may be too broad in it’s specification, and this is reflected by the lack of fit of the six-factor model (raw *χ*^2^ = 3442.63, df = 579, p < 0.001; *χ*^2^/*df* = 5.946; GFI = 0.839; RSMEA = 0.067). Exclusion of the PPD items from the PAPA instrument substantially improved the model fit (see details in results section) so it was decided that a five-factor model (excluding PPD) might represent a more appropriately scoped model to measure parental perceptions about antibiotics.

### Procedure

This study was cross-sectional and employs a previously developed [32], and preliminary validated instrument [33]. Ethical clearances were obtained from Queensland University of Technology in Australia (Ethical approval number: 1200000022) and the Department of Development and Planning in the Ministry of Education in the Eastern Province in Saudi Arabia (Ethical approval number: 33505889). The Arabic questionnaire was distributed to parents of children younger than 12 years old in primary schools in the Eastern Province of Saudi Arabia in September 2012 to January 2013. Attending these meetings is considered a social obligation in Saudi Arabia, thus making the sample a representative one of the Saudi population, adding to the likelihood of external validity of the results. Participants’ consent was implied in the return of the completed questionnaire as shown in the questionnaire’s cover page.

### Instrument development

The PAPA instrument was developed [32] using a content evaluation panel of expert from Australia and Saudi Arabia; the panel of experts were used to conduct the brainstorming process [38]. The instrument’s items were derived from relevant literature, followed by a three-round Delphi Process using content experts. Experts included in preliminary development step came from areas such as: paediatrics, infectious diseases, epidemiology, family medicine, psychology and counselling, and social sciences.

### A *priori* model

After the development of the instrument [32], parallel analysis and Exploratory Factor Analysis (EFA) using principal axis factoring were conducted to determine the number and nature of the underlying factors in the instrument [33]. Six factors were produced from the analysis: knowledge and beliefs, behaviors, sources of information, adherence, awareness about antibiotics resistance, and parents’ perception regarding doctors’ prescribing behaviors. Also, the instrument’s reliability was established with Cronbach’s alpha = 0.78. The constructs produced in the priori model coincide with the constructs contextually available in the relevant literature [33].

An Oblique (Promax) model was chosen after comparing principal axis factoring models with orthogonal (Varimax) and oblique rotations. The latter of these two models was clearly more realistic and revealed substantial correlations among many of the factors [33].

### Statistical analysis

After conducting the EFA analysis using Statistics Package for Social Sciences (SPSS v19: [39]), the resulting constructs from the EFA using Principal Axis Factoring [33] were validated using Confirmatory Factor Analysis (CFA) in AMOS and Stata/SE v12. Results suggested that only five out of the six initial factors should be included in the final CFA model (see above). This study assesses the CFA using a different dataset from the one used in EFA (sample size *n* = 1111).

We initially tried fitting our CFA model using Maximum Likelihood Estimation, but noticed that Generalized Least Squares provided a superior fit. The model fit was evaluated using the Goodness of Fit Index (GFI; [40]), Root Mean Square Error of Approximations (RMSEA; [41]), and the raw and modified *χ*^2^ fit statistics. It is important to note that the raw and modified *χ*^2^ are usually upwardly biased with sample size [42,43] and are included here only for convention. GFI evaluates the model fit by measuring the fit between an estimated model and the observed covariance matrix [40]. A GFI greater than 0.9 is considered a good fit [44]. The RMSEA evaluates the model fit by assessing how well an unknown but optimally chosen parameter estimates fit the population covariance matrix [42] and an RMSEA value of less than 0.06 suggests a good model fit [41].

To establish there was no bias in the pattern of missing values, missing values were analysed using Little’s MCAR test [45] to determine if the missing values are missing completely at random. Only 1.3% of the data was missing, and Little’s MCAR test [45] showed missing values were missing completely at random (p-value = 0.446). The frequency of missing values ranged from 0.4 to 3.3%. Expectation Maximization Technique [40] was used to impute missing values for the purpose of CFA, with all discrete variable imputations rounded to the nearest integer. The internal consistency of the PAPA scales was assessed using Cronbach’s alpha.

To evaluate Convergent validity, the Average Variance Extracted (AVE) for each construct was evaluated against its correlation with the other constructs. Where AVE was larger than the construct’s correlation with other constructs, then Convergent validity was considered to be confirmed [46]. Discriminant validity was established where Maximum Shared Variance (MSV) and the Average Shared Squared Variance (ASV) were both lower than the Average Variance Extracted (AVE) for all the constructs [47].

The total score for each subscale was computed using the loadings for each item produced from the CFA. Each subscale was defined according to what it measures: (1) Knowledge and Beliefs [KB] measures the parents’ knowledge and beliefs about the appropriate use of antibiotics; (2) Behaviours [B] measures the parents’ appropriate behaviours regarding the use of antibiotics; (3) Seeking Information [SI] measures the extent to which parents are proactive in educating themselves about antibiotics from various sources; (4) Antibiotic Adherence [AD] measures the parents’ adherence to appropriate antibiotic doses; and (5) Awareness about antibiotic resistance [ABR] measures the parents’ awareness about antibiotic resistance.

A higher score in the ‘Knowledge and Beliefs’ scale means better knowledge regarding antibiotic use; a higher score in the ‘Behaviors’ scale means better judicious behavior regarding antibiotics use; a higher score in the ‘Adherence’ scale means better adherence to appropriate antibiotic doses; a higher score in the ‘Seeking Information’ scale means more eagerness to seek health-related information; and finally a higher score in the ‘Awareness about Antibiotics Resistance’ scale means better awareness regarding antibiotics resistance. All items worded negatively were reverse coded for the purpose of analysis.

## Results

**Figure 1:**
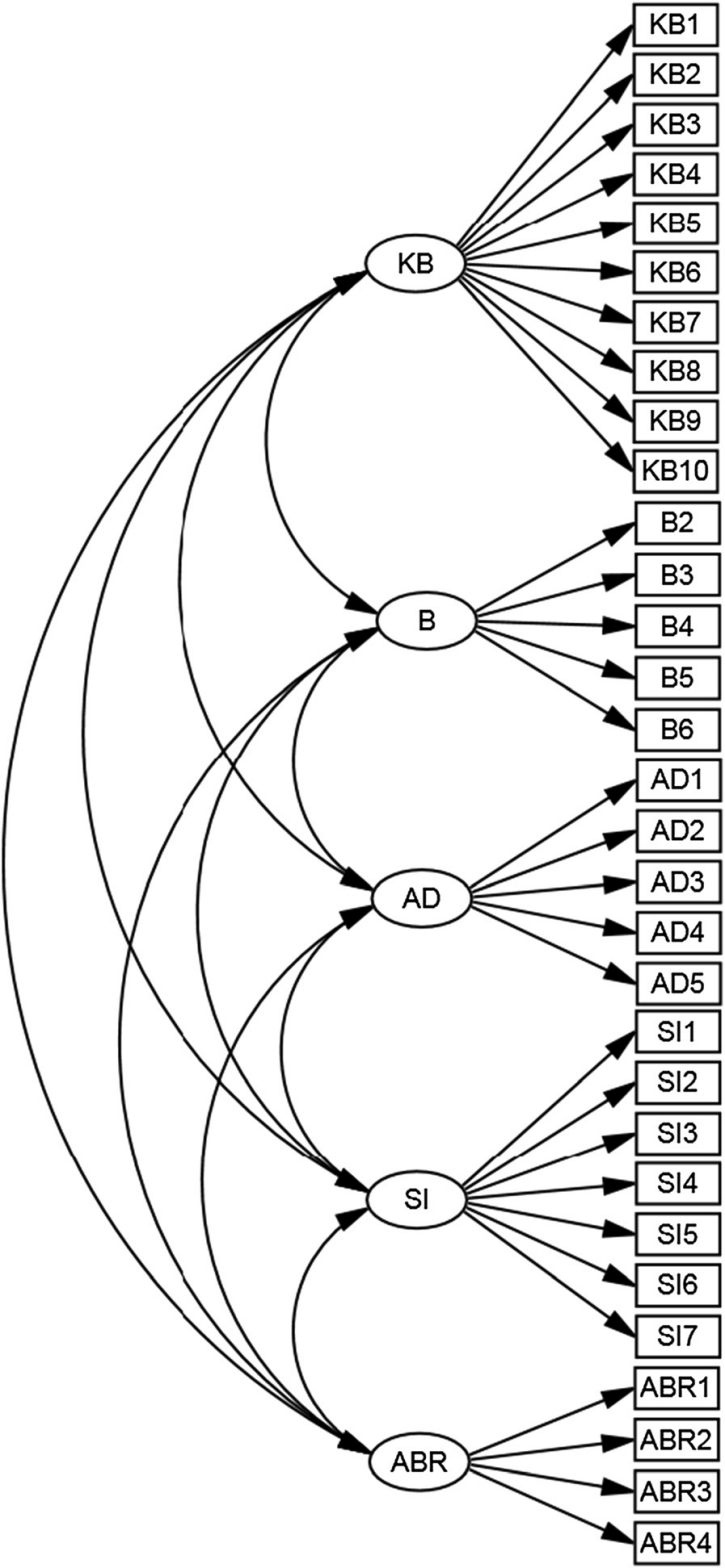
Confirmatory Factor Analysis model.

The measurement model fit using CFA is shown in Figure 1. The model fit the data adequately with a good GFI (GFI = 0.915) and RMSEA (RMSEA = 0.047). The raw *χ*^2^ is 1470.334 and *χ*^2^/*df* is 3.484 with p-value < 0.0001. However, the raw and scaled *χ*^2^ are highly influenced by the sample size [42]. To test this hypothesis, we performed a post hoc analysis by randomly selecting 50% of the sample and rerunning the model; the new raw and scaled *χ*^2^ are 875.123 and 2.074 respectively. Counter-intuitively, the *χ*^2^ reduced dramatically with the reduction of sample size; this concurs with reports from other studies [43].

The means and standard deviations of the individual items in the instrument are presented in Table 1, where 1 is for ‘Strongly disagree’ or ‘never’ and 5 is for ‘strongly agree’ or ‘always’ depending on the nature of the question.

**Table 1:** EFA and CFA Loadings

**Items**	**Loadings**		**Mean (SD)**	**Items**	**Loadings**		**Mean (SD)**
	**β**	***u***			**β**	***u***	
KB1	-0.646	-0.627	2.8 (1.2)	B1	^a^	-0.443	1.6 (1.0)
KB2	-0.367	-0.631	3.2 (1.2)	B2	-0.617	-0.809	1.7 (1.0)
KB3	-0.292	-0.461	3.3 (1.2)	B3	-0.609	-0.534	1.8 (1.1)
KB4	-0.264	-0.574	3.1 (1.1)	B4	-0.825	-0.874	1.8 (1.1)
KB5	-0.660	-0.687	3.2 (1.1)	B5	-0.318	-0.426	1.6 (1.0)
KB6	-0.679	-0.613	2.9 (1.2)	B6	-0.375	-0.623	1.6 (1.0)
KB7	-0.719	-0.672	2.6 (1.2)	AD1	-0.415	-0.716	1.9 (1.0)
KB8	-0.669	-0.749	3.3 (1.1)	AD2	-0.772	-0.902	2.5 (1.3)
KB9	-0.591	-0.628	3.5 (1.0)	AD3	-0.697	-0.517	2.3 (1.2)
KB10	-0.405	-0.594	3.6 (1.0)	AD4	-0.508	-0.697	2.6 (1.3)
SI1	0.427	0.472	2.7 (1.2)	AD5	-0.306	-0.465	1.7 (0.9)
SI2	0.812	0.759	2.8 (1.2)	ABR1	0.468	0.444	3.8 (1.1)
SI3	0.660	0.621	2.3 (1.0)	ABR2	0.572	0.446	3.3 (1.0)
SI4	0.824	0.773	2.6 (1.1)	ABR3	0.587	0.674	3.8 (1.0)
SI5	0.830	0.789	2.6 (1.1)	ABR4	0.186	0.446	3.7 (1.0)
SI6	0.657	0.556	3.3 (1.1)	ABR5	^b^	0.422^c^	
SI7	0.281	0.479	2.6 (1.2)				

Standardized betas (β) from the Confirmatory Factor Analysis and loadings (*u*) from the Exploratory Factors Analysis for the items in the instrument.

All βs in the model are significant at the 0.001 level.

^a^The item was significantly correlated with its factor but had to be removed because it did not load contextually on the construct.

^b^Item was removed from the CFA model because it was not significant.

^c^Items associated with negative loadings in the EFA were reversed scored to get the positive loadings in CFA.

*Abbreviations*: *KB* Knowledge and beliefs, *B* Behaviors, *SI* Seeking Information, *AD* Adherence, *ABR* Awareness about Antibiotics Resistance.

All items in the model loaded significantly at the 0.001 level of significance on their respective factors with standardized betas ranging from 0.19 to 0.83 as shown in Table 1. Out of the ten possible inter-factor correlations, four were significant (Table 2). Furthermore, no substantial cross-loadings (beta > 0.35) were observed in either the EFA [33], nor the CFA.

**Table 2:** Inter-factor correlations

	**Knowledge and beliefs**	**Seeking information**	**Adherence**	**Behaviors**
Seeking information	0.079			
Adherence	0.329*	0.086		
Behaviors	0.212*	0.143	0.332*	
Awareness about antibiotics resistance	-0.154	0.196*	-0.138	0.015

*Significant correlation (p<0.05).

The construct ‘Awareness about antibiotics resistance’ showed only moderate to low internal consistency due to the inclusion of the item that asks about the use of antibiotics to treat bacterial infections. This item exhibited quite a low loading on its respective factor (β = 0.19) but it was included in the model since the item was considered to measure a very important aspect in the ABR scale and this item was shown to have statistically significant association with its factor (β_ABR4_: 0.186, p < 0.001).

The AVE of the constructs in the study were measured and compared to the inter-factor correlations [46]. Preliminary evidence of convergent validity was determined when the AVE of each construct was higher than its correlation with other constructs. While discriminant validity of the PAPA scale was preliminarily determined by assessing the Maximum Shared Variance (MSV) and the Average Shared Squared Variance (ASV), both were found to be lower than the Average Variance Extracted (AVE) for all of the constructs in the scale [47]. Convergent and Discriminant validities results are available in Table 3.

**Table 3:** Convergent and Discriminant validities assessment

**Scales**	**AVE**	**MSV**	**ASV**
Knowledge and beliefs	0.308	0.108	0.046
Behaviors	0.335	0.110	0.044
Sources of Information	0.451	0.038	0.018
Antibiotics Adherence	0.321	0.110	0.061
Awareness about antibiotics resistance	0.231	0.038	0.020

*AVE* Average Variance Extracted.

*MSV* Maximum Shared Variance.

*ASV* Average Shared Squared Variance.

Common Method Bias was evaluated using Harman’s single factor test [48], which determines if the majority of the variance can be explained by a single factor. Common method bias occurs if there is a systematic source of measurement error [49]. In our model, the variance of a single factor was 18.36% indicating there is no common method bias.

## Discussion

The aim of this study was to validate the Parental Perceptions of Antibiotics scales. After producing 36 items from the EFA, CFA was conducted to test the validity of these items. Only 31 items were included in a 5-factor model for the CFA. The five remaining items did not fit the factor structure because: (1) a 3-item factor suggested by the EFA, Parents’ perception about doctors prescribing behavior, led to a poor specification for the initial CFA model; (2) one of the items from the ‘Awareness about antibiotics resistance’ construct was removed because it did not load significantly on its respective factor; and (3) one of the items in the ‘behaviors’ construct was removed since it measured attitudes rather than behavior, and also, another item in the same factor measured a similar aspect, but this latter item was worded to reflect behavior rather than attitude. The resulting 5-factor structure was confirmed as adequately fitting the data. The five factors were: *Knowledge and Beliefs*, *Behaviors*, *Seeking Information*, *Antibiotic adherence*, and *Awareness about antibiotic resistance*.

Our initial inclusion of a subscale to measure parental perceptions regarding doctors prescribing behaviour was revealed to have no place in the PAPA instrument. We still believe this is an important aspect to parental use/misuse of antibiotic in their children, but it may need to be measured separately.

The internal consistency of the individual factors is high except for one factor that demonstrated moderate-low internal consistency. The moderate-low internal consistency of the factor ‘Awareness about antibiotics resistance’ is due to the inclusion of an item with a low, but significant, loading. The item was included because it measures an important aspect.

When parents were asked about the use of antibiotics for common cold, 36% stated that the use of antibiotic was appropriate, while 47% agreed in other studies [50]. Almost half of the sample believed that antibiotics cure children with common cold faster; this coincides with other studies [51,52]. In the study, 43% of the parents believed that antibiotics cure all types of infections including viral, fungal, and bacterial; this concurs with other studies with similar results [53]. In addition, 69% of parents agreed that antibiotics cure bacterial infections. When this aspect was measured in other studies, it produces similar results [52]. These measures of knowledge and beliefs coincide with other studies.

Most parents in the study show good-to-moderate awareness about antibiotic resistance; similar results have been documented in other studies [50,53]. More than half of the parents in our study (56%) indicated that they expect medication (including an antibiotic) when they visit the doctor for their child’s common cold. This result contrasts to the findings in other studies where only about 10% of the parents expected medication (including antibiotics) to treat the common cold [51,52]. This difference in the expectation of antibiotics between the Saudi population and those considered in other studies might be related to the parents’ knowledge or other parental psychosocial factors. Consequently, conducting studies similar to this one will inform researchers on the public’s current knowledge and other psychosocial factors related to the parental use of antibiotics in children and thus target these risk factors.

Approximately 33% of the parents in the study get their health-related information from books and scientific literature, 16% from family and/or friends, and 28% from the Internet. Larson et al. (2006) [53] measured the patient’s sources of information as well, and found that 44% get their health- related information from books and scientific literature, 36% from family and friends, and only 8% from the Internet. The difference in the frequency of getting health- related information from the Internet between our study and Larson et al. (2006) [53] is probably due to temporal variability; where in more recent times, is more accessible, and the usage of the internet to obtain information is now more culturally ingrained.

Some correlations were found within the psychosocial factors in the present study. For instance, parents’ knowledge and beliefs scale was correlated with antibiotics adherence, which is similar to results from other studies [54]. However, the absence of validated instruments for measuring constructs underlying antibiotic use means there is little empirical evidence regarding theory, making present theory in this area, somewhat speculative. In addition, some of the correlations identified in the present study seem contextually sensible. For instance, the positive association between antibiotic adherence and behavior, or the more proactive a parent is seeking of health-related information, the higher their awareness about antibiotic resistance.

The questionnaire used in this study also measures the following criteria: (1) parents’ demographics characteristics such as: gender, number of girls and boys in the family, health training, age, employment status and education levels, geographical background, when they moved to the Eastern Province, and monthly income; and (2) child health-related history including the number of cold episodes and antibiotics used for the youngest child during the last year (ranging from never to more than 6 times a year), and whether any of the children in the family has ever had a serious infectious disease or a chronic disease.

A validated instrument that measures the psychosocial constructs underpinning antibiotic use will allow the investigation of two important sets of relationships. First, what are the parental characteristics associated with the knowledge and behavior relating to antibiotic use (and misuse) in children? Second, how do the various PAPA scales relate to parental practice in terms of administering antibiotics to their children? Further studies are needed to evaluate the PAPA scales (i.e. Knowledge and beliefs, Behaviors, Adherence, Seeking information, and Awareness about antibiotic resistance) against antibiotic consumption.

### Limitations

Since this is the first instrument of its kind to have been fully validated, there are no gold standards to evaluate criteria against it. Criterion related validity cannot be established for this instrument. Also, at this stage of the instrument’s development, we have little idea of its generalizability to other populations. Further studies of the psychometric properties of the PAPA scales in other populations is needed to fully construct validate this instrument.

## Conclusion

This is the first study to attempt a comprehensive psychometric validation of an instrument that measures the psychosocial constructs underlying parental use of antibiotics in their children. An instrument with a 5-factor structure, the PAPA scales, shows strong potential for construct validity. The effectiveness of the PAPA instrument in other populations needs to be established, thereby allowing the investigation of risk factors of antibiotic overuse in populations across the world. Discovering the factors influencing antibiotic use will assist decision-making processes with regards to the best interventions and policy formulations targeted to reduce antibiotic overuse within the community. This, in turn, may reduce the burden of antibacterial resistance, in turn leading to a decrease in the burden of severe infectious diseases caused by antibacterial resistance strains.

## Abbreviations

PAPA: Parents perceptions on antibiotics; URTI: Upper respiratory tract infection; KB: Knowledge and beliefs; B: Behaviors; AD: Adherence; SI: Seeking information; ABR: Awareness about antibiotic resistance; CFA: Confirmatory factor analysis; EFA: Exploratory factor analysis; GFI: Goodness of fit index; RMSEA: Root mean square error of approximations; AVE: Average variance extracted; MSV: Maximum shared variance; ASV: Average shared squared variance.

## Competing interests

The article processing fees are paid by Queensland University of Technology, Australia. There are no other competing interests relating to this research.

## Authors’ contributions

AA and CH substantially participated in the conception and design of the study, and performed the statistical analysis and interpretation. AA and AY conducted data acquisition and AA drafted the manuscript. XH, JS, AY and CH helped to draft the manuscript and revising it critically for important intellectual content. All authors read and approved the final manuscript.

## Pre-publication history

The pre-publication history for this paper can be accessed here:

http://www.biomedcentral.com/1471-2458/14/73/prepub

## Supplementary Material

Additonal file 1 Items in the PAPA scales. Click here for file
