# Supplementary material for: Assessing the construct validity and reliability of the parental perception on antibiotics (PAPA) scales
Source: BMC Public Health. 2014 Jan 23;14:73. doi: 10.1186/1471-2458-14-73 (PMC3909352; doi:10.1186/1471-2458-14-73)
Supplement: Additonal file 1 — Items in the PAPA scales. [file 1471-2458-14-73-S1.docx]

**Additional file 1. Items of the PAPA instrument:**

**Factor 1: Knowledge and beliefs**

[KB3] *Antibiotics treat viral infections*

[KB4] *Antibiotics can cure ALL types of infections (viral, bacterial, & fungal).*

[KB5] Antibiotics are helpful in treating common cold among children

[KB6] *My child will be sick for a longer time if he/she doesn't receive an antibiotic for cough, cold, or flu symptoms*

[KB7] *If my child has a cold or cough, it is best to get an antibiotic to get rid of it*

[KB8] *Children with common colds get better faster when antibiotics are given*

[KB9] *In the past antibiotics have cured my child's cold symptoms*

[KB10] *when I visit the doctor for my child’s common cold, I expect prescription for medication including antibiotics*

**Factor 2: Behaviors**

[B2] *I get my child's antibiotics from the pharmacy without a prescription*

[B3] *I generally store antibiotics at home for when they are needed*

[B4] *In the past, I have given my child an antibiotic without a prescription when he/she had a high temperature for a few days*

[B6] *In the past, I have changed doctors when my doctor did not prescribe antibiotics for my child*

**Factor 3: Adherence**

[AD2] *If my child gets better I can reduce the dose of antibiotics*

[AD3] *If my child’s condition is mild I would give the antibiotic according what I see is suitable for to his/her condition*

[AD4] *In the past, I have stopped giving my child an antibiotic because he/she felt better*

[AD5] *It is not important to follow antibiotics doses strictly*

**Factor 4: Seeking information**

[SI2] *I get my health-related information from books and/or scientific literature*

[SI3] *I get my health-related information from family and/or friends*

[SI4] *I get my health-related information from the Internet*

[SI5] *I get my health-related information from Media (TV, Radio, newspapers)*

[SI6] *I get my health-related information from previous experience*

[SI7] *I get my health-related information from the pharmacist*

**Factor 5: Awareness about antibiotics resistance**

[ABR2] *Some germs are becoming harder to treat with antibiotics*

[ABR3] *Some germs can become resistant to antibiotics if they are taken in inadequate doses*

[ABR4] *Antibiotics treat bacterial infections*
